# Advances in nanomaterials for the diagnosis and treatment of ischemic heart disease

**DOI:** 10.1186/s11671-026-04464-2

**Published:** 2026-02-12

**Authors:** Qinglu Jiang, Feijie Zhou, Yanman Li, Chengfeng Wang, Shan Wang, Houxiang Hu, Zaiyong Zheng

**Affiliations:** 1 Department of Cardiology, People ’s Hospital of Dayi County, Chengdu, 611330 China; 2 Department of Cardiology, Affiliated Hospital of North Sichuan Medical College, No.1, Mao Yuan Nan Road, Nanchong, 637000 China; 3 Department of Cardiology, Langzhong People’s Hospital, Langzhong, 637400 China; 4 Department of Cardiology, Panzhihua Central Hospital, 34 Yikang Street, Middle Section of Panzhihua Avenue, Dong District, Panzhihua, Sichuan 617000 China; 5 Key Laboratory of Medical Electrophysiology, Basic Medicine Research Innovation Center for Cardiometabolic Diseases, Southwest Medical University, Luzhou, China

**Keywords:** Bibliometric analysis, Myocardial infarction, Nanotechnology, Diagnosis, Therapy, Cardiac tissue engineering, Biosensors

## Abstract

Ischemic heart disease (IHD) remains a leading cause of mortality worldwide. Despite timely reperfusion therapy, myocardial ischemia can still lead to ventricular remodeling, arrhythmia, and heart failure, contributing to the high prevalence of cardiovascular disease. Nanomaterials provides innovative technical methods and tools for precise clinical diagnosis and effective treatment through nanoscale control of materials and biological interactions. In this study, bibliometrics have been harnessed to explore the region and topic distribution characteristics of nanotechnology in the field of ischemic cardiomyopathy. The key directions in this field contain cardiac tissue engineering, biosensor, targeted drug delivery and molecular imaging. China and USA are the leading contributors in this filed. Recent literature demonstrates rapid growth in nanomaterial-based strategies and their broad application in preclinical models of IHD. However, only a limited number of nanomedicine products have progressed to clinical approval. This review summarizes the current advances in nanotechnology for IHD, highlights remaining challenges, and discusses future directions to facilitate clinical translation.

## Introduction

Ischemic heart disease (IHD) is a leading cause of mortality worldwide, with myocardial infarction representing one of its most common and severe acute manifestations. Myocardial infarction (MI) occurs when reduced coronary blood supply results in cardiomyocyte death, typically presenting with chest pain and impaired cardiac function [1]. The onset is often abrupt, and in the absence of timely reperfusion, a substantial proportion of cardiomyocytes in the infarcted region may undergo necrosis. Since cardiomyocytes are terminally differentiated with minimal regenerative capacity, lost cells are eventually replaced by fibroblasts. At the same time, diverse stimuli trigger complex extracellular matrix remodeling, which in turn promotes ventricular remodeling and increases myocardial stiffness. This process contributes to left ventricular dilation, impaired cardiac function, and ultimately the development of heart failure, severely affecting prognosis and quality of life. According to the World Health Organization, cardiovascular diseases caused 17.9 million deaths in 2019, accounting for 32% of global mortality—with 85% of these deaths attributed to heart disease and stroke [2]. Myocardial infarction is thus a major cause of premature death worldwide, and its poor regenerative potential frequently results in post-infarction ventricular remodeling and subsequent heart failure.

Nanomaterials, defined as substances with particle sizes between 1 and 100 nm, exhibit unique physicochemical properties owing to their reduced dimensions and increased surface area. These include high surface-to-volume ratios, distinctive optical behavior, and novel electrical and thermal conductivity. Nanotechnology refers to the design and synthesis of highly customized materials at the nanoscale by manipulating individual atoms and molecules. As many biomolecules in the human body fall within this size range, nanotechnology offers the unprecedented opportunity to construct diagnostic tools and therapeutic agents at the molecular level.

By leveraging these unique properties, nanotechnology provides new strategies to overcome the limitations of current approaches for IHD diagnosis and treatment. To date, its applications in IHD have been concentrated in four major areas: bioengineering, disease diagnosis, drug delivery, and imaging [3]. This field therefore holds tremendous potential for clinical translation. In this review, we apply bibliometric and visualization methods to systematically analyze and present the evolving landscape of nanotechnology in ischemic heart disease. Our aim is to enhance comprehensive understanding, stimulate integrative thinking, and provide new perspectives for researchers in this rapidly developing area.

## Materials and methods

Literature search was conducted in the Web of Science Core Collection [4], using topic terms related to both nanotechnology and ischemic heart disease. The search strategy was defined as:

TS = (nanoparticle* OR nanomaterial* OR nanodot* OR “quantum dot*” OR nanosphere* OR nanorod* OR nanofiber* OR nanotube* OR nanosheet* OR nanocomposite* OR nanodevice* OR nanocluster* OR nanotechn* OR nanocarrier* OR nanowire* OR nanoliposome* OR nanoemulsion* OR nanocrystal* OR nanogel* OR nanoconjugate* OR nanodiamond*) [3] AND TS = (“myocardial infarct*” OR “heart infarct*” OR “acute coronary syndrome” OR “coronary heart disease” OR “ischemic heart disease” OR “myocardial ischaemia” OR “myocardial ischemia”) AND LA = (English) AND DT = (Article OR Review).

The publication time frame was set from the earliest records available up to September 1, 2025. A total of 2,976 articles were retrieved. The full records and cited references of these publications were exported in plain text format, and the resulting data were processed using VOSviewer (version 1.6.18.0) and Pajek (version 6.01) for knowledge-mapping and visualization analyses.

## Results

### Annual publication trends, distribution of the most productive countries/regions and institutions, and research directions

**Fig. 1:**
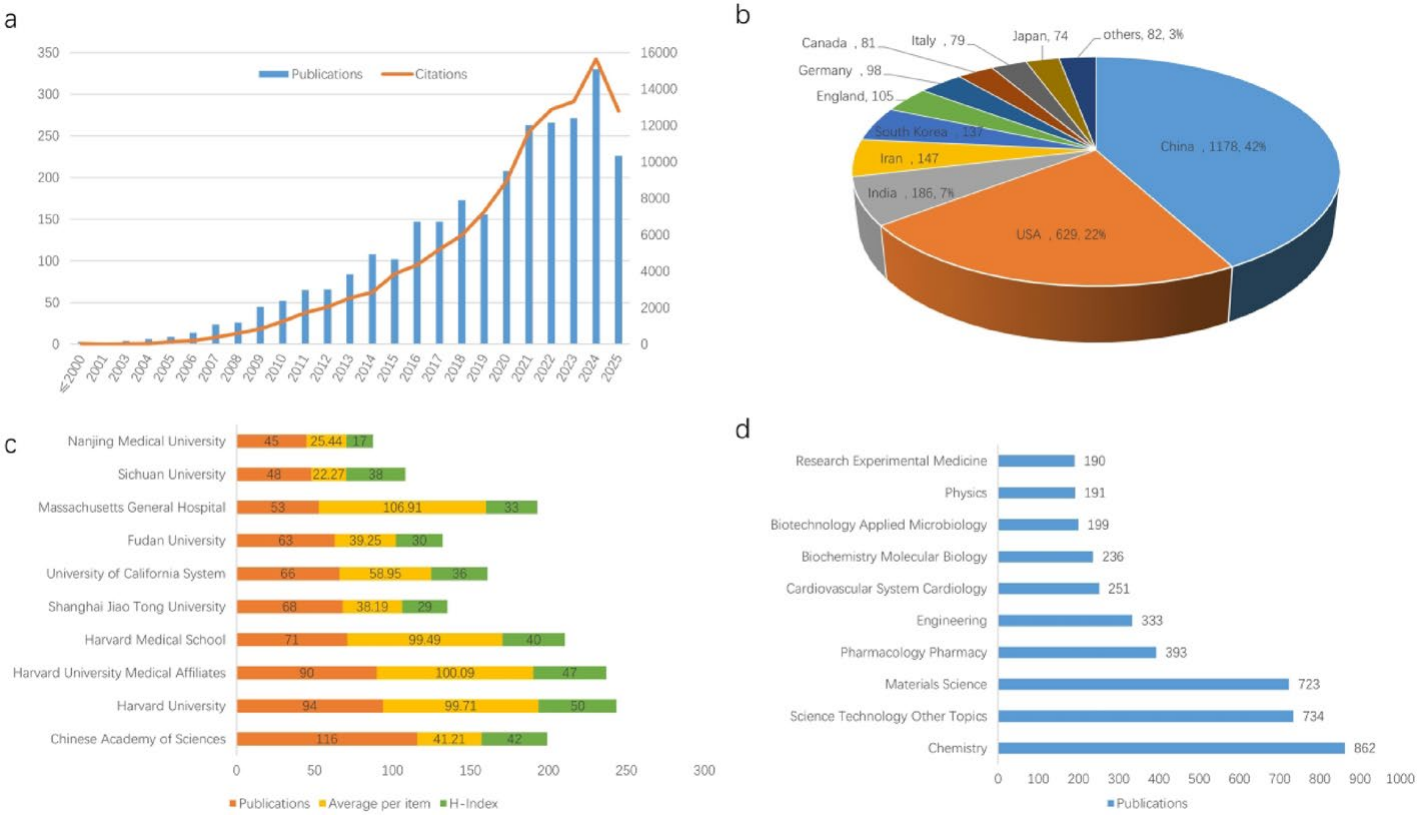
Distribution of publications on nanotechnology in ischemic heart disease. **a** Annual distribution of publications and citations. **b** Geographic distribution of publications by country/region. **c** Top 10 research institutions by publication volume, with the number of publications, average citations per article, and h-index. **d** Distribution of publications across research fields.

The literature published in the field of nanotechnology applied to myocardial infarction showed a steady annual increase between 2000 and 2013, followed by a rapid rise from 2014 to 2024. Meanwhile, the number of citations also grew in parallel with publication volume and time progression (Fig. 1a). These results indicate that the field of nanotechnology in ischemic heart disease has attracted broad attention from scholars worldwide, with an increasing number of researchers and institutions participating in related studies.

The leading countries by publication volume were China, followed by the United States, India, Iran, South Korea, England, Germany, Canada, Italy, Japan (Fig. 1b). China published 1178 articles (42% of the total), and the United States with 629 articles (22%). At the institutional level(Fig. 1c), the top three contributors were the Chinese Academy of Sciences (116 articles), Harvard University (94 articles) and Harvard University Medical Affiliates (90 articles), highlighting their extensive research in this area (Fig. 1c). In terms of average citations per article, Massachusetts General Hospital, Harvard University Medical Affiliates and Harvard University ranked highest, with averages of 106.91, 100.09, and 99.71 citations per paper, respectively, reflecting the strong impact of their work. The H-index further underscores research influence: Harvard University(H-index:50), Harvard University Medical Affiliates (H-index:47), and the Chinese Academy of Sciences(H-index:42), indicated that these institutions have produced not only a large number of publications but also high-quality research in this field.

An analysis of research topics provides insights into the developmental trends of the field. As shown in Fig. 1d, the main research areas were chemistry, multidisciplinary science and technology, materials science, pharmacology and pharmacy, engineering, cardiovascular system and cardiology, biochemistry and molecular biology, biotechnology and applied microbiology, physics, research and experimental medicine. Among them, the top three fields were chemistry (862 publications), multidisciplinary science and technology (734 publications), and materials science (723 publications). These results indicate that most nanotechnologies still focused on the materials themselves, and only a few nanotechnologies have been applied in clinical practice (Fig. 1d).

### Co-citation analysis of journals

**Fig. 2:**
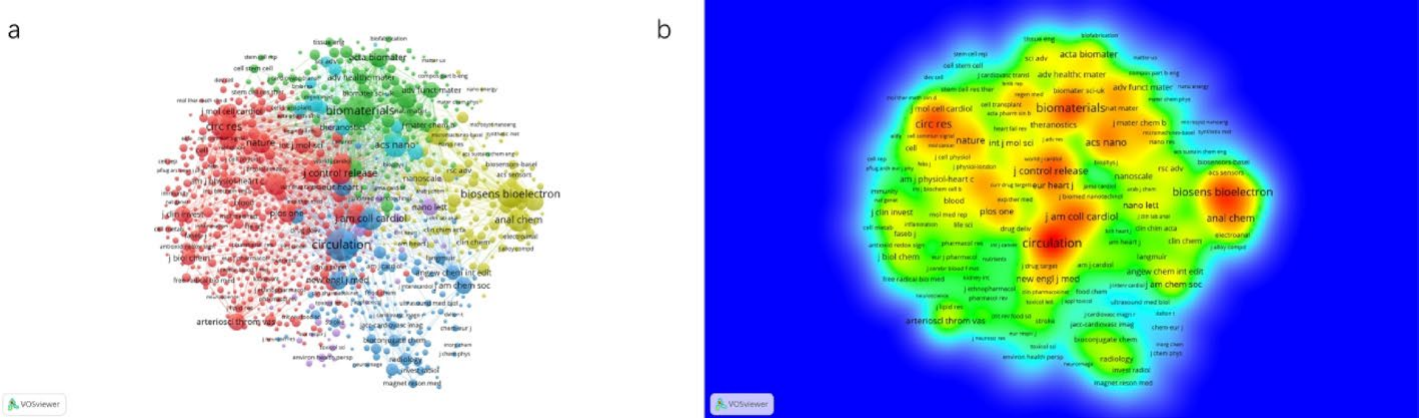
Co-citation analysis of journals. **a** Network visualization of co-cited journals (six clusters). **b** Density visualization of co-cited journals

Co-citation analysis helps identify the most influential sources cited within a research domain, thereby revealing the core journals and thematic field. In this field, the five most highly co-cited journals were *Circulation* (6,200 citations), *Biomaterials* (5,151 citations), *Biosensors* & *Bioelectronics* (3,807 citations), *Circulation Research* (3,475 citations), *Journal of the American College of Cardiology* (2,513 citations) (Fig. 2). Most of these journals are closely related to nanotechnology and cardiovascular science. Cluster analysis revealed six main application domains of nanomaterials in ischemic heart disease: stem cells and regenerative medicine (red cluster), analytical chemistry and sensor technology (yellow cluster), biomaterials and biomedical engineering (green cluster), cardiovascular and medical imaging (purple cluster), materials science and nanotechnology (blue cluster).

### Research hotspots in the application of nanotechnology to ischemic heart disease

**Fig. 3:**
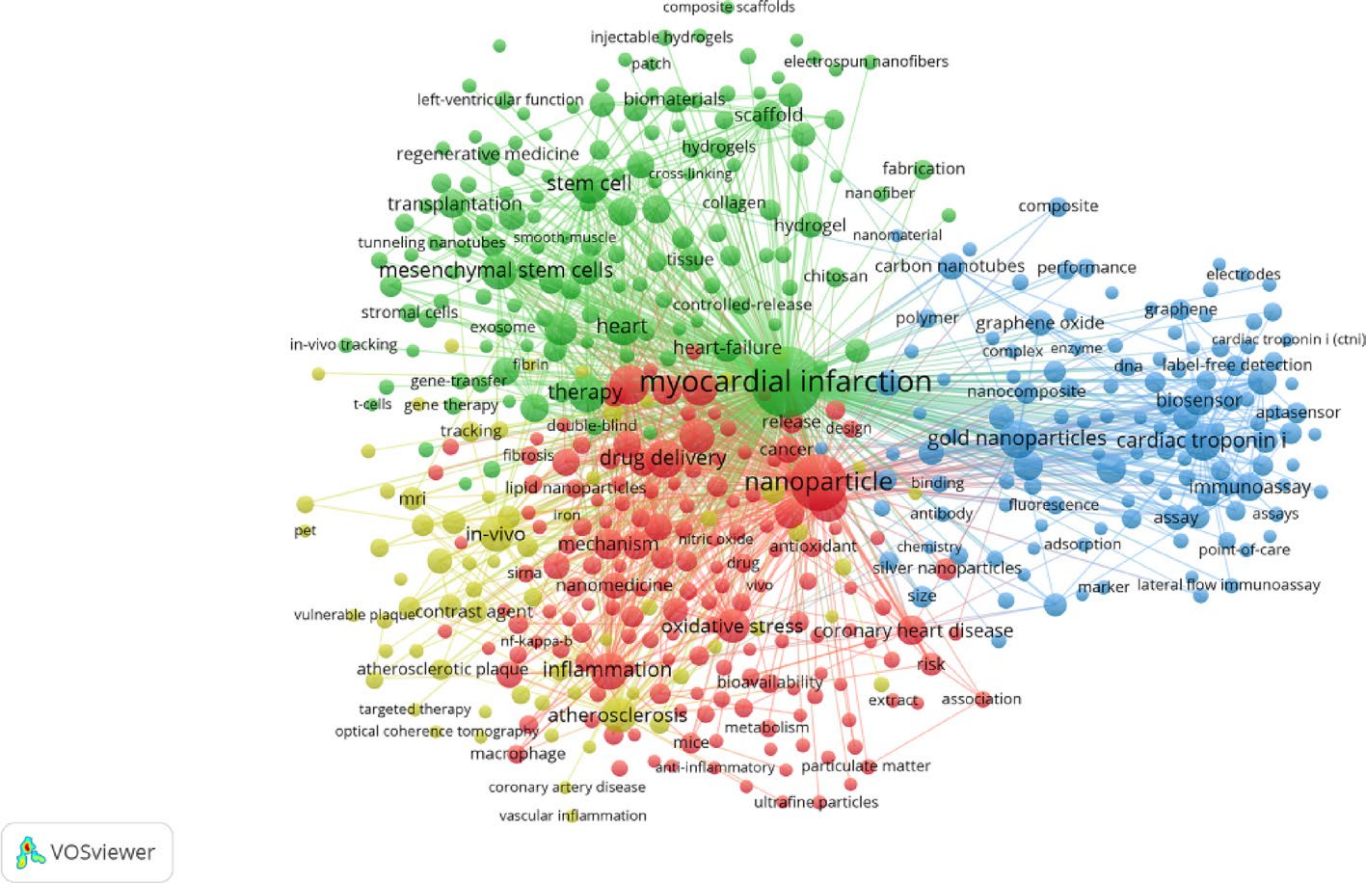
Keyword co-occurrence analysis of publications on nanotechnology in ischemic heart disease

As the concise indicators of core content, keywords can reveal research hotspots through co-occurrence analysis—providing valuable guidance for tracking the evolving focus of this field. VOSviewer analysis identified 10,219 unique keywords, of which 494 appeared more than 10 times. The top 10 most frequent keywords were: myocardial infarction (1,399 times), nanoparticle (731 times), delivery (256 times), gold nanoparticles (240 times), drug delivery (239 times), stem cell (227 times), cardiovascular disease (225 times), inflammation (216 times), cardiac troponin I (213 times) and therapy (211 times) (Fig. 3). Cluster analysis further indicated that nanotechnology applications in ischemic heart disease primarily focus on four areas: disease mechanisms and therapeutics (red cluster), which include inflammation, apoptosis, oxidative stress, drug delivery, bioavailability, endothelial dysfunction, and anti-inflammation; tissue engineering (green cluster), including angiogenesis, cardiac regeneration, heart regeneration, mesenchymal stem cells, cardiac progenitor cells, extracellular matrix, hydrogel, scaffolds, exosomes, and so on; nanodiagnostics and biosensors (blue cluster), including biosensors, electrochemical biosensors, aptamers, cardiac troponin I, biomarkers, graphene oxide, quantum dots, gold nanoparticles, etc.; and imaging (yellow cluster), including atherosclerosis, acute coronary syndromes, myocardial infarction, imaging, MRI, CT, OCT, PET, drug-eluting stents, contrast agents, etc.

##  Discussion: applications of nanotechnology in ischemic heart disease

As shown in Fig. 3, current literatures about nanotechnology in ischemic heart disease focus on therapeutics, tissue engineering, biosensors and imaging. In this section, we provide a comprehensive review of each topic with representative examples.

### Applications of nanotechnology in therapeutics

In recent years, therapeutic strategies aimed at repairing damaged myocardium have rapidly progressed, including cell therapy, genetic engineering, anti-inflammatory and pro-angiogenic agents, and cardiac-specific delivery approaches [5]. Although promising, these interventions face significant translational challenges due to nonspecific systemic distribution, inadequate accumulation in target tissues, and the need for invasive procedures for targeted cardiac delivery. Nanomaterials, with their favorable biocompatibility and tunable biological properties, represent ideal carriers for efficient, controlled drug delivery to infarcted myocardium (red cluster in Fig. 3). Nanomedicines can also be engineered to release drugs specifically at lesion sites by exploiting local differences in temperature, enzyme activity, or pH. Additionally, external stimuli such as ultrasound, magnetic fields, or infrared irradiation can trigger controlled release. For example, Lim et al. successfully delivered magnetic nanoparticles to the heart using magnetic targeting [6]. Hybrid magnetoliposomes—which combine magnetic nanoparticles and liposomes—have also been widely investigated due to their responsive release under external magnetic fields, holding great promise as multifunctional drug delivery systems [7]. Given these advantages, nanomedicine approaches have been explored to target each major step of IHD pathogenesis, from atherosclerotic plaque formation to post-infarction scar remodeling.

#### Atherosclerosis (plaque formation)

At the initial stage of IHD, risk factors such as hyperlipidemia and endothelial injury promote the development of atherosclerotic plaques. Nanotechnologies have been employed to reduce plaque burden and stabilize lesions. For instance, a self-assembly nanoparticle platform was used to deliver IL-10 mRNA into atherosclerotic lesions, which elevated the anti-inflammatory cytokine IL-10 and ultimately decreased lipid deposition in plaques [8]. Beyond drug delivery, other nanotechnology-based approaches such as nanozymes, cholesterol scavengers, and photothermal ablation have been developed to combat atherosclerosis. Cerium oxide nanoparticles (CeO₂), with unique redox-catalytic properties, have been utilized as nanozymes to scavenge reactive oxygen species (ROS) in plaques in ApoE^−/−^ mice, CeO₂ nanozymes significantly reduced total plaque area [9, 10]. Another approach is to enhance cholesterol removal from plaques. Gong et al. synthesized nanosponge-like liposomes functionalized with Annexin V to target atherosclerotic lesions, these nanosponges selectively absorbed cholesterol and led to plaque reduction [11]. Similarly, cyclodextrin-based nanoparticles (~ 10 nm in size) have been shown to bind excess cholesterol, significantly promoting cholesterol efflux from plaque tissue without notable systemic toxicity [12]. Periodic mesoporous silica nanoparticles have also been reported to selectively absorb LDL cholesterol from the bloodstream, aiding plaque [13]. Luo et al. designed a phospholipid-based high-density lipoprotein-mimicking nanoparticle (composed of DPPC and DSPE-PEG_2000) that promoted cholesterol efflux via biliary pathways while sparing normal arterial walls [14]. Another innovative strategy leverages the optical and thermal properties of nanomaterials for plaque ablation. Tu et al. used near-infrared laser irradiation to heat osteopontin-targeted polydopamine nanoparticles within plaques, achieving an approximate 13% reduction in plaque area [15].

#### Thrombosis (plaque rupture and clot formation)

Rupture of an atheromatous plaque triggers platelet aggregation and thrombus formation, acutely occluding the coronary artery and leading to myocardial ischemia. Nanotechnology has been harnessed to improve thrombolytic therapy and limit clot formation. Zhang et al. developed a thrombolytic nanocomplex by conjugating a novel urokinase to multi-walled carbon nanotubes (MWCNTs) functionalized with chitosan and an Arg-Gly-Asp peptide. This construct enhanced thrombus targeting, extended the duration of fibrinolysis, and reduced systemic side effects [16]. In a similar vein, Guo et al. created phenylboronic acid-functionalized nanocarriers co-loaded with an antioxidant (protocatechualdehyde) and the thrombolytic drug tissue plasminogen activator (tPA). When delivered to the occluded coronary artery, this nanotherapy successfully dissolved thrombi [17]. These examples illustrate how nanocarriers can be engineered for targeted delivery of thrombolytics, providing rapid recanalization while minimizing off-target effects.

#### Cardiomyocyte death

If ischemia persists for a few minutes, myocardial cells undergo irreversible injury, including various forms of cell death. Nanotherapeutic strategies have been explored to protect cardiomyocytes and reduce cell death in the acute phase of myocardial infarction. For example, Yu et al. designed a silica nanoparticle carrier to deliver microRNA-24 into ischemic cardiomyocytes, which significantly inhibited apoptosis after MI [18]. In another study, Fan et al. formulated Poly(lactic-co-glycolic acid) [18] nanoparticles loaded with anti-apoptotic and pro-survival agents (CHIR99021 and fibroblast growth factor 1). In both mouse and pig MI models, these nanoparticles markedly reduced cardiomyocyte death and decreased infarct size by 20–30% [19]. Such nanomedicine approaches aim to salvage jeopardized myocardium during AMI, limiting the extent of necrosis by delivering cytoprotective molecules directly to at-risk cells.

#### Post-infarction inflammation

Necrotic and dying cells release damage-associated molecular patterns (DAMPs) that activate innate immune receptors, triggering a robust inflammatory response in the infarcted heart. Nanocarriers have been utilized to modulate this post-MI inflammation by delivering anti-inflammatory therapeutics specifically to the injured myocardium. Somasuntharam et al. delivered an anti-inflammatory DNAzyme via gold nanoparticles injected into the heart, which significantly reduced pro-inflammatory tumor necrosis factor-alpha (TNF-α) levels after AMI [20]. Likewise, various studies report using nanomaterials to target anti-inflammatory drugs to the infarct zone [21, 22]. For instance, calcium carbonate nanoparticles loaded with colchicine, when administered into the myocardium, significantly decreased inflammatory mediators such as C-reactive protein, TNF-α, and interleukin-1β after AMI [23]. Among widely used nanocarriers, biodegradable PLGA nanoparticles have shown particular promise in cardiac inflammation. Sun et al. demonstrated that resveratrol-loaded PLGA nanoparticles attenuated myocardial inflammation and injury in an isoproterenol-induced AMI model, yielding significant cardioprotective effects [24]. By damping excessive inflammation, these nanotherapies create a more favorable environment for subsequent healing.

#### Granulation tissue formation and scar remodeling

In the later stages of ischemic cardiomyopathy, populations of fibroblasts, endothelial cells, and progenitor cells infiltrate the infarct and form granulation tissue. This healing phase is accompanied by extensive extracellular matrix (collagen) deposition, which eventually leads to scar tissue formation replacing the necrotic myocardium. Nanotechnology-based therapies have been employed to improve healing and prevent detrimental ventricular remodeling at this stage [25, 26]. For example, a peptide-based hydrogel co-assembled with the bioactive peptide V1-Cal was applied to MI hearts and found to significantly decrease cardiac fibrosis, thereby limiting scar formation [27]. In another study, a platelet-inspired nanocell coated with prostaglandin E2 and cardiac stromal cell-secreted factors was used to activate endogenous cardiac stem/progenitor cells, which mitigated adverse cardiac remodeling and improved heart function post-MI [25]. Additionally, Varshosaz et al. developed an injectable thermosensitive hydrogel scaffold (derived from adipose extracellular matrix) embedded with atorvastatin-loaded lipid nanocapsules and gold nanoparticles; this combination therapy promoted cardiomyocyte proliferation and myocardial regeneration in the infarcted area [28]. By targeting fibrosis and encouraging regeneration, such nanomaterial-based interventions aim to strengthen the infarct border zone and restore cardiac tissue, ultimately improving outcomes in post-infarction remodeling.

### Applications of nanotechnology in cardiac tissue engineering

With advances in stem cell biology, materials science, and tissue engineering, cardiac tissue engineering has emerged as a promising strategy for disease modeling, drug development, and myocardial repair. Given the limited regenerative capacity of cardiomyocytes, the shortage of donor organs, and the poor retention and survival of transplanted stem cells, engineered cardiac tissues may provide a feasible alternative therapy [29]. This approach integrates cells from suitable sources with scaffold biomaterials to construct grafts that replace damaged cardiac tissue and restore or improve cardiac function [29]. Primary cells and stem cells remain the main cellular sources for cardiac tissue engineering [30]. Stem cells not only differentiate into cardiomyocytes but also exert therapeutic effects via paracrine signaling, modulating the extracellular matrix microenvironment [31]. Due to their excellent physicochemical properties and biocompatibility, nanomaterials have been increasingly incorporated into scaffolds, patches, and injectable constructs in cardiac tissue engineering.

Novel engineered biomaterials enhance stem cell survival in ischemic regions by providing structural support, releasing cytokines, regulating cell–material interactions, and establishing a favorable microenvironment—thereby promoting myocardial regeneration and repair [32]. The cardiac extracellular matrix (ECM), primarily composed of collagen and glycoproteins, plays an essential role in cell growth, differentiation, and maintenance of cardiac architecture and function [33]. The high tunability and scalability of nanotechnology enable the synthesis of complex bioengineered scaffolds. Nanomaterial-based scaffolds can replicate native ECM by forming intricate three-dimensional structures, modifying surface properties, incorporating specific adhesion molecules, or delivering bioactive factors, thus providing mechanical support, conductive environments, and biological cues for cells [34]. By varying the bioactive components carried by nanoscaffolds, cellular behaviors such as migration, proliferation, adhesion, and differentiation can be modulated [35].

Electrospinning offers efficient nanoscale synthesis of fibers with diverse compositions, mimicking ECM complexity. Chen et al. designed an adipose-derived mesenchymal stem cells patch fabricated from electrospun cellulose nanofibers modified with chitosan/silk fibroin multilayer membranes. This construct reduced fibrosis, inhibited apoptosis, promoted angiogenesis, improved cell survival, and limited ventricular remodeling [36]. Similarly, Sridhar et al. showed that gold nanoparticle-functionalized nanofibers enhanced mesenchymal stem cell proliferation and cardiomyogenic differentiation [37]. Collagen, a key ECM component, was mimicked by Liu et al. using poly(l-lactic acid) scaffolds to culture embryonic stem cell–derived cardiovascular progenitor cells, enabling in vitro construction of cardiac tissue structures [38]. The interconnected porous architecture facilitated cell adhesion, migration, and differentiation.

Normal cardiomyocyte contraction relies on intercellular communication and electrical coupling. Reestablishing these connections is critical for restoring conduction in infarcted myocardium [39]. Nair et al. incorporated gold nanoparticles into porcine gallbladder ECM to fabricate conductive nanocomposite scaffolds that promoted angiogenesis and cell proliferation within infarcted tissue [40]. Conductive nanomaterials such as graphene and carbon nanotubes transmit mechanoelectrical and electrochemical signals to improve intercellular communication and scaffold conductivity, thereby supporting functional tissue formation [32, 41]. Shokraei et al. developed biocompatible carbon nanotube/polyurethane nanofiber scaffolds with enhanced cell–cell communication [42]. Conductive nanofiber patches combining electrical conductivity and flexibility have also gained attention as effective tissue-engineering constructs. Mousa et al. fabricated electrospun conductive nanofiber patches mimicking the biological and mechanical properties of native ECM while providing strong conductivity for electrically responsive tissue, enhancing adhesion and proliferation [43].

Nanofibers can also act as carriers of bioactive molecules. Growth factors encapsulated in polymeric micro- or nanocarriers promote differentiation, homing, and anti-apoptotic activity. Zhu et al. incorporated human vascular endothelial growth factor-165 (hVEGF_165_) plasmids into hyperbranched polyamidoamine gene vehicles to transfert skeletal myoblasts for use in myocardial repair [44].

The anisotropic organization of cells and ECM underlies cardiac structure and function, yet reproducing this orientation remains a key challenge. Techniques such as electrospinning and 3D bioprinting are increasingly applied. Hu et al. developed micropatterned, electroactive, and biodegradable polymer membranes from poly (glycerol sebacate) and aniline trimer, mimicking myocardial anisotropy, electrophysiology, and mechanics [45]. López-Canosa et al. engineered a microphysiological system combining electrospun fibers and electrical stimulation via a microfluidic platform, enhancing cell alignment, differentiation, and maturation [46]. Similarly, 3D bioprinted nanomaterial scaffolds have been employed to guide stem cell differentiation and cardiomyocyte alignment [47]. Tijore et al. fabricated gelatin hydrogel microchannels via 3D bioprinting, demonstrating improved mesenchymal stem cell alignment, cardiac lineage differentiation, and rhythmic contractility [48]. Wu et al. proposed a 3D hybrid scaffold incorporating hydrogel shells with oriented conductive nanofiber yarn networks (polycaprolactone, silk fibroin, and carbon nanotubes). This system precisely controlled cell orientation across layers, promoting cardiomyocyte maturation [49].

Integration of cell reprogramming with bioengineering platforms has enabled engineered cardiac organoids, offering in vitro models that recapitulate human cardiac structure and function. Organoids are 3D assemblies of tissues or specific cell types encapsulated within natural or synthetic ECM scaffolds [50]. Innovative 3D bioprinting methods have produced vascularized organoids applicable to regenerative medicine, drug screening, and disease modeling. Zhang et al. used 3D bioprinting to generate endothelialized microfibrous scaffolds, showing that engineered endothelium supported the formation of aligned cardiomyocytes capable of spontaneous, synchronous contraction [51]. Furthermore, embedding nanoelectronic devices within organoids allows long-term electrophysiological monitoring. Li et al. demonstrated that stretchable mesh nanoelectronics could be integrated into 3D organoids with minimal interference in growth and differentiation, enabling systematic observation of cardiac organoid electrophysiological dynamics during development [52].

Although nanotechnology has greatly advanced the field of cardiac tissue engineering and revealed substantial potential for treating ischemic heart disease, several major challenges still remain. First of all, the long-term safety of many nanomaterials is not yet well understood, raising concerns about possible cardiotoxicity and chronic adverse effects [53]. Second, although some tissue-engineering designs appear promising in theory, their performance in vivo often falls short of expectations. For example, the benefits of stem-cell-based cardiac patches are now thought to arise mainly from the secretion of extracellular vesicles, as the transplanted cells themselves rarely survive for long [36, 54]. Third, the ventricular wall is a highly complex, anisotropic, and constantly beating structure, and current tissue-engineered constructs cannot fully replace native myocardium or match its dynamic biomechanical demands. Moreover, many reported constructs require direct myocardial injection or even open-chest delivery, which further increases procedural difficulty in clinical settings. At the same time, tissue-engineered products are inherently complex combination therapies, making their regulatory pathway even more challenging and slowing their translation into routine clinical practice.

### Applications of nanotechnology in biosensors

Biomarkers are biological molecules that reflect physiological or pathological states and are widely used for disease diagnosis, identification of high-risk individuals, therapeutic decision-making, and prognosis evaluation [55]. Cardiac troponin is the principal clinical biomarker for screening and diagnosing myocardial infarction [56]. However, conventional assays for troponin are often limited by narrow detection ranges and prolonged turnaround times, underscoring the need for highly sensitive detection methods.

Biomarker detection typically involves two processes: target recognition and signal detection. Recognition relies on the specific binding of antigens and antibodies or aptamers to target molecules. Following complex formation, spontaneous or induced signals (acoustic, optical, or electrical) are generated and captured by devices to quantify biomarker concentration or status [57]. The unique physicochemical properties of nanomaterials—arising from surface effects [58], macroscopic quantum tunneling [59], quantum size effects [60], volume effects [61], and dielectric effects [62]—offer broad prospects for developing high-performance electrochemical and electrochemiluminescent biosensors (blue cluster in Fig. 3). Compared with traditional methods, nanosensors feature high sensitivity, high throughput, rapid analysis, and portability (see Table 1). Their large surface-to-volume ratios allow loading of multiple antibodies for simultaneous detection of several biomarkers, while optical and electrical properties enhance signal amplification and improve sensitivity.

In electrochemical biosensors, electrodes are modified with nanomaterials to amplify recognition signals and convert them into electrical readouts for qualitative or quantitative analysis [63]. Such modifications significantly enhance electron transfer and adsorption capacity for bioactive molecules, thereby shortening detection time. Among these, electrochemical aptamer-based sensors for cardiac troponin I (cTnI) show particular promise. Aptamers—single-stranded nucleic acids or peptides with high binding affinity—offer advantages over antibodies including ease of synthesis, chemical stability, pronounced conformational changes, and facile functionalization [64, 65]. For example, Sun et al. developed an enzyme-free electrochemical aptasensor using magnetic metal–organic framework nanocatalysts and DNA tetrahedron-based dual aptamer probes. The dual probes Tro4 and Tro6, immobilized on screen-printed gold electrodes, enabled highly enhanced cTnI capture with a detection limit as low as 16 pg/mL [66].

Metal nanomaterials also enhance biosensors by labeling or adsorbing bioactive molecules, and by directly participating in electrode reactions as reactants or catalysts. This improves current peaks, reduces redox potential, and amplifies signal strength [67]. Surya et al. designed a label-free aptamer field-effect transistor sensor using gold nanoparticle-modified Co₃O₄ nanorods combined with single-walled carbon nanotube (SWCNT) layers for cardiac troponin T (cTnT) detection. The synergy of gold nanoparticles and Co₃O₄ nanorods improved sensitivity by 250%, achieving a detection limit of 0.1 mg/mL [68].

Graphene quantum dots, composed of sp²-hybridized carbon atoms, exhibit unique size-dependent optical and electrochemical properties, making them attractive platforms for biosensing. Karaman et al. fabricated a sandwich-type electrochemical immunosensor using nitrogen and boron-doped graphene quantum dots with Ce-doped SnO₂/SnS₂ (Ce-SnO2/SnS2) for signal amplification, achieving an ultra-low cTnI detection limit of 2.00 fg/mL in plasma samples [69].

Nanotechnology also enables the conversion of chemical signals from antigen–antibody interactions into amplified optical signals for methods such as electrochemiluminescence assays, fluorescence immunoassays, and surface plasmon resonance immunoassays. For instance, Wang et al. constructed a sandwich-type electrochemiluminescent immunosensor using molybdenum disulfide@cuprous oxide–silver nanoparticles (MoS₂@Cu₂O–Ag) for electrode modification and cerium-doped zinc oxide@nitrogendoped graphene quantum dots (Ce: ZnO–NGQDs) for signal amplification. The incorporated nanomaterials provided excellent conductivity, biocompatibility, and large surface area, enhancing electron transfer rates. As a result, the sensor achieved a linear range of 10 pg/mL–100 ng/mL and a detection limit of 2.90 fg/mL [70]. Collectively, nanomaterial-based biosensors demonstrate remarkable improvements in sensitivity, specificity, and throughput for electrochemical detection, positioning them as key technologies for future diagnostics.

The high sensitivity and signal-amplification capabilities of nanotechnology have greatly accelerated the discovery of emerging biomarkers, including miRNAs, lncRNAs, and extracellular vesicles (EVs). For instance, Li et al. developed a spherical nucleic acid–based catalytic probe capable of simultaneously detecting two AMI-associated miRNAs—miR-499 and miR-133a—using nothing more than a smartphone as the portable readout device [71]. In addition, nanotechnology has also offered new insight into traditional biomarkers. Tiambeng et al. engineered peptide-functionalized superparamagnetic nanoparticles to efficiently capture low-abundance cTnI, enabling a top-down nanoproteomic analysis that revealed substantial diversity in cTnI proteoforms across different cardiovascular conditions [72].

**Table 1:** Nanobiosensors for AMI biomarkers

Biosensor type	Target	Nanomaterial / Strategy	Detection limit	Linear range	Assay time	Reference
Electrochemical immunosensor	NT-proBNP	Bi_2_WO_6_/Ag_2_S nanoparticles with graphene oxide / polydopamine	0.03 pg/mL	0.1 pg/mL-100 ng/mL	Not specified	[73]
Electrochemiluminescence	NT-proBNP	CuS on reduced graphene oxide	0.12 pg/mL	0.5 pg/mL-20 ng/mL	Not specified	[74]
Naked-eye detection kit	cTnI	Colorimetric ELISA strip	0.32 ng/mL	0.32–200 ng/mL	70 min	[75]
Electrochemical biosensor	cTnI	Atomic layer-deposited NbS_2_ nanoflakes on carbon fiber	0.32 fM (0.007 ng/L)	1 fM-0.1 nM	120 min	[76]
Conductometric nanowire	cTnI, CK-MB, BNP, Myoglobin	Single polyaniline nanowires with monoclonal antibody functionalization	cTnI: 250 fg/mL; CK-MB: 150 fg/mL; BNP: 50 fg/mL; Myo: 100 pg/mL	fg/mL to ng/mL	Few minutes	[77]
Optical plasmonic sensor	cTnI	Gold nanoledge arrays with DNA aptamer	0.084ng/mL	Not specified	Not specified	[78]
Surface plasmon resonance	cTnT	Gold nanorods	7.6 fg/mL	7.6 fg/mL-0.9 mg/mL	About 50 min	[79]
Paper-based high-sensitivity vertical flow assay	cTnI	Gold nanoparticle	0.2pg/mL	Not specified	15 min	[80]
Photoelectrochemical	cTnI	Bi_2_O_2_S nanosheets	–	0.2 pg/mL-10 ng/mL	Not specified	[81]
Fluorescence nanosensor	miR-499	MoS_2_ nanoshee	381.78 pM	0.1 nM-13.3 nM	30 min	[82]
Electrochemiluminescence	miR-126-3p	mPEG-SH modified Au nanoparticles	–	1 nM-1 fM	Not specified	[83]
Surface-enhanced resonance raman scattering	cTnI	Streptavidin-coated gold nanostar	0.403 ng/L	0.5 ng/L-50,000 ng/L	Not specified	[84]
Magnetic dual-aptamer electrochemical sensor	cTnI	N-ZIF-67@PBA	0.31 fg/mL	10 fg/mL-1 ng/mL	Not specified	[85]
Naked-eye detection kit	cTnI	AuNP	0.12 ng/mL	0.5–50 ng/mL	15 min	[86]
Surface-enhanced raman spectroscopy	CK-MB, Mb and cTnI	Gold-silica-gold alternately layered pyramidal plasmonic metasurface	CK-MB 0.05ng/mL; Mb 3.8ng/mL ; cTnI 7.0pg/mL;	CK-MB 0.3-33.3ng/mL; Mb 3.3-1333ng/mL cTnI 10-9009pg/mL	Not specified	[87]

### Applications of nanotechnology in imaging

Conventional cardiac imaging differentiates normal and diseased tissues by leveraging their distinct responses to X-rays, ultrasound, and magnetic fields, which is crucial for the diagnosis and management of myocardial infarction. An ideal imaging modality should not only delineate cardiac and great-vessel morphology, size, position, and intracavitary anatomy, but also provide information on valvular and cardiac function, hemodynamics, myocardial perfusion and metabolism, and tissue characteristics. In cardiac imaging, contrast agents are essential for distinguishing myocardium from the cardiac chambers, improving contrast between normal and pathological tissues and thereby enhancing detection rates [88]. Traditional contrast agents, however, suffer from rapid clearance, poor targeting, and potential toxicity, whereas nanoparticles can boost resolution and amplify signals [89]. Molecular imaging refers to the visualization and quantification of pathogenic molecular or cellular alterations at the molecular level [90]. For example, positron emission tomography (PET) with ¹⁸F-fluorodeoxyglucose (FDG) enables noninvasive visualization of glucose uptake in tumor tissues. The concept of nanoscale dimensions perfectly aligns with the fundamental requirements of molecular imaging. These strategies have also been applied to the IHD. Accordingly, advanced molecular imaging probes based on functional nanomaterials—combined with state-of-the-art imaging techniques—offer a reliable route to improve the accuracy of clinical cardiac imaging.

MI typically results from thrombotic occlusion following rupture or erosion of an unstable coronary plaque. Visualizing thrombi and ischemia are therefore pivotal for MI diagnosis and treatment. In routine computed tomography (CT) and magnetic resonance imaging (MRI), thrombus identification often relies on visualization of reduced intraluminal flow rather than direct thrombus imaging, risking missed detection of fragments or distal emboli. By coupling nanomaterials with targeting ligands, CT/MRI agents can concentrate on thrombi, enabling precise localization and direct visualization. During myocardial ischemia and after coronary revascularization, X-ray angiography reports luminal stenosis but cannot directly grade lesion severity, whereas nanotechnology-enabled probes can resolve details of the ischemic penumbra [88]. Moreover, multimodal imaging refers to the integrated use of two or more imaging techniques to obtain structural, functional, and molecular information about the human body [91]. With the advantage of nanomaterials that can carry multiple contrast agents, multimodal molecular imaging enables different imaging modalities (such as CT, MRI or ultrasound ) to be performed using a single contrast agent.

## Magnetic resonance imaging (MRI)

Cardiac MRI offers high spatial resolution, precise tissue localization, no ionizing radiation, and relatively short imaging windows. Iron oxide–based contrast agents (iron oxide nanoparticles) possess intrinsic magnetic properties and good biocompatibility, showing great promise in MI imaging [92]. Inflammation drives cardiovascular pathogenesis, with macrophages exerting both injurious and reparative roles; quantifying macrophage activity aids assessment of myocardial inflammation [93]. Ultrasmall superparamagnetic iron oxide (USPIO) nanoparticles change local proton relaxation and apparent proton density, resulting in shortened T2/T2* relaxation and low signal intensity, thereby changing local MR characteristics. Their accumulation in plaque macrophages enables noninvasive monitoring of plaque progression and responses to anti-inflammatory therapy [94]. For example, Moonen et al. reported using T2 mapping MRI to quantify USPIO uptake in atherosclerotic plaques near the murine heart [95]. Gadolinium-based agents are widely used clinically. Gd³⁺, with strong paramagnetism among lanthanides, enhances T1 and can reduce T2 signals [96]. Cheng et al. showed that gadolinium diethylenetriamine pentaacetic acid (Gd-DTPA) could be loaded into nanocomposites with improved T1 relaxivity and selectively accumulated in inflamed endothelium overexpressing P-selectin, increasing MRI detectability while lowering cytotoxicity [97].

### Ultrasound imaging

Ultrasound is convenient, inexpensive, noninvasive, and provides substantial tissue penetration, making it widely used clinically. Optimally distributed ultrasound contrast agents can markedly improve image quality. Common agents include microbubbles, nano-bubbles, gas-producing nanoparticles, and echogenic liposomes [98]. Wang et al. prepared anti-Vascular cell adhesion molecule (VCAM)-1 nano-scale ultrasound microbubble agents via a hyperbranched self-assembly method, enhancing visualization of vulnerable atherosclerotic plaques and supporting molecular ultrasound diagnostics [99]. Echogenic liposomes combine lamellar lipid structures that capture gas to form ultrasound-responsive microbubbles, making them attractive for sonographic imaging [100]. Kim et al. demonstrated that nitric-oxide–loaded echogenic liposomes, activated by ultrasound, improved endothelial permeability and facilitated delivery of anti-VCAM-1–targeted ELIP across the arterial wall to inflamed sites, thereby enhancing plaque visualization [101]. In addition, decorating nano-contrast agents with specific ligands enables targeting of disease-related cellular antigens [102]. For example, Zhou et al. engineered CNA35-labeled perfluoropentane (CNA35-PFP) liposomal nanoparticles that target type I collagen on fibroblasts within fibrotic myocardium, enabling targeted, real-time ultrasound imaging of myocardial fibrosis [103].

### Computed tomography (CT)

CT provides tomographic images with high temporal and spatial resolution using X-rays and computerized reconstruction. Iodinated compounds strongly attenuate X-rays and are the most common CT contrast agents, but their nonspecific distribution and short circulation limit performance. Functional nanoparticle contrast agents can prolong circulation and confer specificity, expanding imaging windows and improving contrast at lower doses [104]. Targeted agents can also enable molecular imaging of specific plaque components, such as macrophages in atherosclerotic lesions. Van Herck et al. used N1177 with multislice CT (MSCT) to detect ruptured plaques in rabbits, showing significantly increased CT attenuation at rupture sites and suggesting utility for early lesion detection in acute coronary syndrome [105]. Iodinated polymeric nanoparticles have been developed as effective contrast agents for spectral photon counting computed tomography (SPCCT), capable of providing enhanced cardiovascular imaging in rats following intravenous administration [106]. In this context, the study detailed the construction of iodinated polymer nanoparticles through the loading of iodine onto a poly(vinyl alcohol) (PVAL) backbone, resulting in a formulation with high iodine content and improved imaging properties. Beyond iodine, nanoparticles based on gold, lanthanides, tantalum, and other elements have been explored as efficient CT agents. Gold nanoparticles, with excellent biocompatibility and strong X-ray attenuation, are widely used; tailoring particle shape and surface ligands yields multifunctional, targeted probes for lesion imaging [107]. Photon-counting CT (PCCT) further enables K-edge imaging- which exploits the abrupt change in X-ray attenuation that occurs when photon energies cross the K-shell binding energy of a specific element- to generate quantitative elemental maps [108]. Si-Mohamed et al. found that PCCT utilizing gold nanoparticle-assisted K-edge imaging could detect and quantify macrophage burden in atherosclerotic rabbit aortas [109].

### Near-infrared fluorescence imaging

Optical imaging employs powerful microscopy and diverse contrast mechanisms for molecular visualization. Fluorescence imaging is noninvasive, highly sensitive, and operationally simple [110]. Engineered fluorescent nanoparticles can be designed as probes responsive to disease-specific biomarkers, enabling targeted imaging of lesions [111]. Early thrombi are small, unstable, widely distributed, and rapidly formed; Near-infrared (NIR) fluorescence offers high spatiotemporal resolution and deep tissue penetration for direct in situ thrombus visualization [112]. Zhang et al. developed a thrombus-targeted NIR probe that markedly improved signal-to-noise and resolution for thrombus imaging [113]. Beyond thrombosis, biofunctional NIR-emissive nanoparticles facilitate in vivo cardiac imaging after AMI. Mateos et al. leveraged fast acquisition and selective targeting with NIR-II (second near-infrared window) nanodots to achieve instantaneous in vivo imaging of acute MI [114]. Ortgies et al. used angiotensin II peptide–functionalized Ag₂S nanodots as NIR probes for infarcted hearts, providing valuable information on the location and extent of myocardial injury to aid identification of the occluded artery and indirect assessment of damage severity [115].

### Positron emission tomography (PET) and single-photon emission computed tomography (SPECT)

PET and SPECT are γ-ray–based modalities with deep penetration, high sensitivity, and short scan times. In the heart and vasculature, inflammatory macrophages exacerbate tissue injury and promote disease; radiolabeled tracers enable targeted organ imaging. Nahrendorf et al. used ⁶⁴Cu–Macrin nanoparticles as PET nanotracers for noninvasive, quantitative assessment of cardiac macrophages [116]. Shafiee Ardestani et al. developed ⁹⁹ᵐTc-dendrimer glucose conjugate (⁹⁹ᵐTc-DGC), which accumulated in myocardium and enabled early, accurate SPECT diagnosis of MI [117].

Given that each modality has distinct strengths and limitations, multimodality molecular imaging using multifunctional nanoprobe platforms can synergize complementary information and improve MI diagnostics [118]. Coronary microthrombi contribute to no-reflow following acute ischemia–reperfusion, yet ultrasound, CT angiography, and MRI often lack molecular specificity for microthrombi. Recent molecular imaging strategies address this gap. Bai et al. designed PLGA-cRGD-PFH-ICG NPs (PLGA: poly(lactic-co-glycolic) acid, cRGD: cyclic arginine-glycine-aspartic acid, PFH: perfluorohexane, ICG: indocyanine green, NPs: nanoparticles) that target thrombi via surface cRGD, encapsulate ICG, and enabled dual photoacoustic/NIR fluorescence imaging in a rat ischemia–reperfusion model [119]. Because plaque rupture precipitates complications such as ischemia and MI, early imaging of atherosclerosis is critical; Mehta et al. showed that ultrasound-responsive cyclodextrin nanoparticles support multimodal imaging and therapy of atherosclerosis [120]. Persistent myocardial ischemia causes irreversible injury, cardiomyocyte death, and contractile dysfunction, underscoring the need for early, accurate diagnosis and monitoring. Chen et al. developed an ischemia-targeted nanoprobe (IMTP-Fe₃O₄-PFH NPs) with enhanced ultrasound (US), photoacoustic(PA), and MRI performance for direct, noninvasive visualization of ischemic myocardium in rats, significantly boosting in vivo US/PA/MR signals and demonstrating feasibility for early detection and (Table 2) image-guided therapy [121].

**Table 2:** Nanoparticle-Enhanced imaging in disease models: strategies and strengths

Imaging modality	Nanoprobe type	Target	Experimental species	Key strengths/ Strategies	Reference
MRI	SPIO nanoparticles	Macrophages in infarcted myocardium	Human	Sensitive inflammation imaging, macrophage tracking	[122]
SPECT/CT	Gold nanoparticles	Macrophage-rich atherosclerotic plaques	Mice	MRI-visible nanoparticles enabling combined imaging and photothermal therapy for atherosclerosis.	[123]
Ultrasound	VCAM-1-targeted nanobubbles	Activated endothelium in plaques	Human endarterectomy specimens	Real-time molecular imaging of inflammation by using Ultrasound-targeted contrast imaging of VCAM-1	[124]
Photoacoustic	Gold nanorods	Macrophages in atherosclerotic plaques	Rabbit	IVPA imaging with Au NPs enables specific macrophage detection in plaques.	[125]
PET	^18^F-labeled polyglucose nanoparticles	Macrophages in infarct and plaque	Primate	Deep-tissue inflammation quantification.	[126]
NIR-II Fluorescence/MRI	NaNdF_4_@NaGdF_4_ + scFv	Atherosclerotic plaques	Mice	Deep tissue optical imaging, high contrast.	[127]
Photoacoustic imaging	DPP-EDOT π-conjugated polymer nanoprobe	Atherosclerotic plaques	Mice	A dual-responsive, lesion-targeted nanoplatform integrating photoacoustic imaging with a three-in-one lipid-lowering therapeutic strategy.	[128]
CT (X-ray)	sAu/GSH-LF	blood vessels	Mice	Develop gold nanoparticle-based X-ray contrast agents to enable long-term, high-resolution imaging of tumor vasculature.	[129]
Photoacoustic (PA) + NIR Fluorescence	Erythrocyte-derived nanoparticles doped with ICG	Atherosclerotic lesions, coronary artery occlusion (post-MI model)	Mice	Combine NETs-based contrast agents with photoacoustic imaging for noninvasive, high-resolution detection of coronary artery inflammation and occlusion.	[130]
MRI (T1-weighted)	Polydopamine NPs doped with arginine + Gd³⁺ + miR-146a	Inflammatory macrophages in plaques	Mice	miRNA delivery + ROS scavenging + MRI imaging.	[131]
Photoacoustic + Ultrasound + CT	CNA35-GNR/PFP@NPs	Collagen I/III in fibrotic myocardium	Rat	Collagen-binding NPs enable US/PA/CT imaging of fibrotic myocardium.	[132]
Photon-counting CT (PCCT), K-edge mode	AuNPs,20-25 nm core diameter, PEGylated	Macrophage-rich atherosclerotic plaques	Rabbit	Gold k-edge PCCT enables simultaneous anatomical and macrophage-targeted molecular imaging in atherosclerosis, even with calcification.	[109]
Photoacoustic (PA) + Fluorescence (FL)	CHP-functionalized albumin nanoparticles (ICG- or PTX-loaded)	Degraded collagen in plaques	Rabbit	CHP-functionalized albumin nanoparticles enable collagen-targeted imaging and therapy of atherosclerotic plaques.	[133]
US/PA/MRI	IMTP-Fe₃O₄-PFH nanoparticles	Ischemic myocardium	Rat	Multimodal US/PA/MR imaging of ischemic myocardium using targeted IMTP-Fe₃O₄-PFH nanoprobes.	[121]
NIR-II Fluorescence	Ag₂S nanodots (biofunctionalized)	Infarcted myocardium	Mice	Fast NIR-II imaging of ischemic myocardium via AngII-functionalized Ag₂S nanodots targeting AT1R.	[114]
Photoacoustic imaging	PA/ASePSD (π-conjugated polymer-based intelligent responsive nanoparticle)	Atherosclerotic plaque	Mice	Cascade-responsive nanoparticle enables targeted imaging and synergistic therapy for early atherosclerosis.	[134]

The most suitable nanomaterials for different imaging technologies are various. For example, superparamagnetic iron oxide nanoparticles are widely used in MRI due to their high T2 relaxivity, which increases image contrast and enables better delineation of ischemic regions [122]. For CT, high-atomic-number materials such as gold or bismuth enhance X-ray attenuation and improve contrast resolution [129]. In addition, nano contrast agents can be functionalized with targeting or therapeutic ligands, which can enhance their accumulation in the heart or enable theranostic applications at the same time.

## Future direction and clinical challenge

Nanomaterials provide powerful new tools for the diagnosis and treatment of IHD and hold promises to alleviate current clinical dilemmas. In biosensing, nanomaterial-based sensors substantially amplify signals and improve sensitivity, thereby widening linear ranges and lowering detection limits for biomarkers such as cTnI and cTnT—offering new avenues for early cardiac diagnostics. Owing to high sensitivity and low background noise, electrochemical detection is the most widely applied; its deep integration with nanotechnology, microfluidics, and related techniques (including screen printing and patterning) is expected to further enhance electrochemical immunosensor performance. Looking ahead, portable, low-cost, point-of-care electrochemical devices leveraging nanomaterials are likely to become increasingly prevalent. Moreover, biosensors built on quantum dots, field-effect platforms, nucleic-acid aptamers, metal nanoparticles, and carbon nanomaterials have exhibited good selectivity and suitable sensitivity, enabling rapid, quantitative, and label-free detection of cardiac biomarkers.

In drug delivery, liposomes, dendrimers, polymeric nanoparticles, and gold nanoparticles can act as carriers to deliver drugs and other therapeutic molecules to injured myocardium via passive or active targeting. As efficient, specific, and controllable intracellular delivery systems, nanocarriers can improve drug stability and aqueous solubility, tailor biodistribution, achieve tissue specificity, and reduce adverse effects. Extracellular vesicles have emerged as lipidic carriers for targeted release of therapeutics and other bioactive cargo, providing superior biocompatibility, lower immunogenicity, and greater plasma stability [135]. Nevertheless, the biocompatibility of nanodrug carriers themselves (and their degradation products), as well as potential cytotoxicity, remain concerns. Current data on pharmacodynamics, organ accumulation, and metabolic pathways are still preliminary; high-throughput characterization and further clinical trials are needed to enable clinical translation.

In imaging, MRI, US, CT, NIR, PET, and SPECT—when combined with nanoparticle probes—can markedly improve diagnostic accuracy and specificity for cardiac applications, yielding clearer and more precise visualization of thrombi and ischemia. Multimodal strategies that synergistically combine modalities can capitalize on complementary strengths to provide a more comprehensive diagnostic picture in MI. Beyond thrombus and ischemia imaging, nanoparticles can also be applied to angiogenesis, blood-pool, and stem-cell imaging [136]. Direct or indirect labeling and tracking of stem cells in vivo remains an active area of research, enabling comprehensive evaluation of stem-cell location, survival, and even differentiation status [137]. Nanoparticles—owing to biocompatibility, simplicity, tunable functionality, and capacity for real-time monitoring—are frequently used for stem-cell labeling, supporting emerging cell-therapy approaches [138].

Despite these advances, only a few nanomedicines have approved for cardiovascular disease, and most of them primarily aim to improve the stability, half-life, bioavailability, or safety profiles of existing drugs [139]. There are several barriers that still hinder current clinical translation. First, long-term safety remains insufficiently understood. Many nanomaterials tend to accumulate in tissues or degrade slowly, and their chronic effects on health are still unclear. More comprehensive preclinical models, standardized toxicological testing, and long-term post-marketing surveillance are needed to fully characterize these risks [140]. The nanomedicines reported in the literature are often made in laboratory conditions using techniques that can’t be scaled up. Developing simple, robust, and cost-effective large-scale manufacturing processes with stringent quality control is critical for their industrial translation. Third, there are substantial regulatory hurdles. The nanoscale confers unique properties that differ from those of conventional materials, complicating risk assessment. In addition, many nanomedicines are multifunctional and compositionally complex (e.g., incorporating multiple active molecules, targeting ligands, and imaging moieties), which poses considerable challenges for existing regulatory frameworks. There is still a lack of harmonized, nano-specific regulatory guidelines, validated characterization assays, and consensus criteria for quality, safety, and performance evaluation.

To overcome these challenges, closer collaboration among basic researchers, clinicians, toxicologists, industrial partners, and regulatory agencies will be essential. Such multidisciplinary efforts can accelerate the development of standardized characterization methods, scalable manufacturing technologies, and science-based regulatory pathways. With these coordinated advances, nanotechnology has the potential to move from proof-of-concept studies toward safe, effective, and widely accessible nanomedicines for patients with IHD.

## Conclusions and outlook

Using bibliometric software such as VOSviewer, we conducted a visualized bibliometric analysis of literature on nanotechnology for the diagnosis and treatment of ischemic heart disease. We reviewed and analyzed publication trends by year, the most productive countries/regions and institutions, research areas, co-cited references, and keyword co-occurrence to summarize advances and emerging directions in this field. From 2000 to 2024, both publication counts and citation frequencies increased steadily, indicating growing scholarly interest and maturation of nanotechnology research in ischemic heart disease. China, the United States, and India contributed the largest number of publications. The top three institutions by publication volume were the Chinese Academy of Sciences, Harvard University, and Harvard University Medical Affiliates; by average citations per article, Massachusetts General Hospital, Harvard University Medical Affiliates and Harvard University ranked highest; and by H-index, Harvard University, Harvard University Medical Affiliates and the Chinese Academy of Sciences led the field. The five most frequently cited journals were *Circulation*, *Biomaterials*, *Biosensors* & *Bioelectronics*, *Circulation Research*, *Journal of the American College of Cardiology*.

Keyword co-occurrence analysis indicated four major hotspots: tissue engineering, diagnosis, therapeutics, imaging. In cardiac tissue engineering, novel nanomaterials enhance cardiomyocyte and stem-cell adhesion and promote favorable morphology and gene expression, thereby improving the microenvironment and enabling functional therapeutic effects. Nanotechnology also supports the development of ECM-mimicking scaffolds, reconstruction of electrical coupling and conduction between cardiomyocytes, and loading of bioactive molecules—expanding options for engineered cardiac repair. Recent studies of cardiac patches, injectable hydrogels, extracellular vesicle–based therapies, and advanced scaffolds have shown promising results in vitro and in vivo [141]. In parallel, multi-material 3D printing of vascular structures, valves, and myocardium aims to recapitulate native cardiac architecture and function, and stem-cell and reprogramming technologies are being used to construct 3D cardiac models. These approaches remain early-stage; accurately building fully functional cardiac analogs will require further investigation.

In summary, research on nanotechnology in ischemic heart disease is advancing rapidly in both depth and breadth. Bibliometric visualization helps the community accurately grasp hotspots and frontiers, providing reliable clues and guidance for further investigations. Nanotechnology holds broad application prospects and substantial potential for the diagnosis and treatment of ischemic heart disease.

## Data Availability

No datasets were generated or analysed during the current study.

## Abbreviations

IHD: Ischemic Heart Disease
AMI: Acute Myocardial Infarction
MRI: Magnetic Resonance Imaging
CT: Computed Tomography
OCT: Optical Coherence Tomography
PET: Positron Emission Tomography
IL-10: Interleukin-10
ROS: Reactive Oxygen Species
LDL: Low-Density Lipoprotein
DPPC: 1,2-Dipalmitoyl-Sn-Glycero-3-Phosphocholine
DSPE-PEG_2000: 1,2-Distearoyl-Sn-Glycero-3-Phosphoethanolamine-Poly(Ethylene Glycol)_2000
MWCNTs: Multi-Walled Carbon Nanotubes
Arg-Gly-Asp: Arginylglycylaspartic Acid
TPA: Tissue Plasminogen Activator
PLGA: Poly(Lactic-Co-Glycolic Acid)
DAMPs: Damage-Associated Molecular Patterns
TNF-α: Tumor Necrosis Factor-Alpha
CRP: C-Reactive Protein
ECM: Extracellular Matrix
hVEGF165: Human Vascular Endothelial Growth Factor-165
cTnI: Cardiac Troponin I
SWCNT: Single-Walled Carbon Nanotube
cTnT: Cardiac Troponin T
EVs: Extracellular Vesicles
Bi2WO6/Ag2S: Bismuth Tungstate/Silver Sulfide Composite
ELISA: Enzyme-Linked Immunosorbent Assay
CK-MB: Creatine Kinase-Mb
BNP: B-Type Natriuretic Peptide
Mb: Myoglobin
FDG: ¹⁸F-fluorodeoxyglucose
USPIO: Ultrasmall Superparamagnetic Iron Oxide
Gd-DTPA: Gadolinium Diethylenetriamine Pentaacetic Acid
VCAM: Vascular Cell Adhesion Molecule
ELIP: Echogenic Liposomes
CNA35-PFP: Cna35-Labeled Perfluoropentane
MSCT: Multislice Ct
SPCCT: Spectral Photon Counting Computed Tomography
PVAL: Poly(Vinyl Alcohol)
PCCT: Photon-Counting Ct
NIR: Near-Infrared
NIR-II: Second Near-Infrared Window
cRGD: Cyclic Arginine-Glycine-Aspartic Acid
PFH: Perfluorohexane
ICG: Indocyanine Green
US: Ultrasound
PA: Photoacoustic
SPIO: Superparamagnetic Iron Oxide Nanoparticles
VCAM-1: Vascular Cell Adhesion Molecule-1
DPP-EDOT: Diketopyrrolopyrrole-Ethylenedioxythiophene (Copolymer)
GSH-LF: Glutathione-Lactoferrin
PEGylated: Polyethylene Glycol-Modified
FL: Fluorescence
IMTP: Integrated Molecular-Targeted Photothermal Therapy
AT1R: Angiotensin Type 1 Receptor
PA/ASePSD: Photoacoustic/Analog Silicon Photomultiplier Detector
